# Preventive Treatment with Methylprednisolone Paradoxically Exacerbates Experimental Autoimmune Encephalomyelitis

**DOI:** 10.1155/2012/417017

**Published:** 2012-11-29

**Authors:** Simone Wüst, Jens van den Brandt, Holger M. Reichardt, Fred Lühder

**Affiliations:** ^1^Institute for Multiple Sclerosis Research, University of Göttingen and Gemeinnützige Hertie-Stiftung, Waldweg 33, 37073 Göttingen, Germany; ^2^Neuroimmunology Branch, National Institute of Neurological Disorders and Stroke (NINDS), National Institutes of Health (NIH), Bethesda, MD 20892, USA; ^3^Institute of Cellular and Molecular Immunology, University of Göttingen Medical School, Humboldtallee 34, 37073 Göttingen, Germany; ^4^Department of Laboratory Animal Science, Medical School, University of Greifswald, Walther-Rathenau-Straße 49a, 17489 Greifswald, Germany

## Abstract

Glucocorticoids (GCs) represent the standard treatment for acute disease bouts in multiple sclerosis (MS) patients, for which methylprednisolone (MP) pulse therapy is the most frequently used protocol. Here, we compared the efficacy of therapeutic and preventive MP application in MOG_35-55_-induced experimental autoimmune encephalomyelitis (EAE) in C57Bl/6 mice. When administered briefly after the onset of the disease, MP efficiently ameliorated EAE in a dose-dependent manner. Surprisingly, MP administration around the time of immunization was contraindicated as it even increased leukocyte infiltration into the CNS and worsened the disease symptoms. Our analyses suggest that in the latter case an incomplete depletion of peripheral T cells by MP triggers homeostatic proliferation, which presumably results in an enhanced priming of autoreactive T cells and causes an aggravated disease course. Thus, the timing and selection of a particular GC derivative require careful consideration in MS therapy.

## 1. Introduction

MP is extensively used for the treatment of acute relapses in MS patients in the clinic [1]. In most cases, the drug is well tolerated when applied at a high dose of up to 2 g/day for a short period of time [2, 3]. However, a slightly higher risk of serious infections was recently reported [4]. Other side effects include metabolic changes, hepatotoxicity, osteoporosis, hypertension, edema, and psychological changes, although these complications are rare and predominantly observed after prolonged application [5, 6].

EAE is a widely employed animal model of MS and often used for the investigation of its pathomechanism as well as for studies concerning drug development [7]. In C57Bl/6 mice, immunization with MOG_35-55_ leads to a chronic disease course, characterized by fulminant inflammation, demyelinating lesions, and subsequent axonal damage [8, 9]. Previously, we have used this model to demonstrate that dexamethasone (Dex) efficiently reduced the clinical symptoms of EAE when given either in a preventive or therapeutic setting [10]. This beneficial effect was accompanied by reduced lymphocyte infiltration into the central nervous system (CNS), induction of apoptosis of peripheral lymphocytes, and reduced T-cell migration to the spinal cord [10]. Additionally, production of proinflammatory cytokines by lymphocytes was reduced after administration of a dissociated GC [11], an effect that was also observed after MP therapy of EAE [12]. Importantly, we had found that MP was less efficient in ameliorating EAE compared to Dex or other fluorinated GC derivatives [13]. Therefore, we here investigated in more detail the characteristics of MP, the most widely used GC derivative in the treatment of MS. We confirmed its clinical efficacy in a therapeutic setting, but it surprisingly enhanced the disease course when administered around the time of immunization. The latter observation was corroborated by histological analyses and could be associated with the induction of homeostatic T-cell proliferation which enhances T-cell priming. Thus, our results indicate that the correct timing of GC therapy might be important.

## 2. Materials and Methods

### 2.1. Mice

C57Bl/6 mice used for EAE induction were purchased from Harlan (Borchen, Germany). Mice constitutively expressing red fluorescence protein (RFP) in all organs including cells of the immune system have been described elsewhere [14]. All animal experiments were approved by the responsible authorities in Lower Saxony (LAVES).

### 2.2. Protocols for EAE Induction and Treatment

EAE was induced as previously described [10]. Briefly, mice were immunized with 50 *μ*g MOG_35-55_ peptide in CFA and 400 ng pertussis toxin. Animals were weighed and scored daily for clinical signs of disease on a scale from 0 to 10 depending on severity; scores were as followed: 0 = normal; 1 = reduced tone of tail; 2 = limp tail, impaired righting; 3 = absent righting; 4 = gait ataxia; 5 = mild paraparesis of hindlimbs; 6 = moderate paraparesis; 7 = severe paraparesis or paraplegia; 8 = tetraparesis; 9 = moribund; 10 = death. 

Mice were treated three times on consecutive days i.p. with methylprednisolone-21-hydrogensuccinat (MP; Urbason solubile, Sanofi-Aventis) at different doses (ranging from 100 mg/kg to 0.8 mg/kg). In the preventive setting, MP administration was started one day before immunization and in the therapeutic setting once the mice had reached an average score of 2-3. Control mice were injected with equal volumes of PBS.

### 2.3. Histological Analysis by Immunohistochemistry

Immunohistochemistry was performed as described previously [10]. Briefly, mice were perfused with PFA, and paraffin-embedded spinal cord sections were stained with an anti-human CD3 (1 : 200; Serotec, Düsseldorf, Germany) or an anti-mouse Mac-3 antibody (1 : 200; BD, Heidelberg, Germany). This was followed by incubation with a secondary biotinylated rabbit anti-rat antibody (1 : 200; Vector Laboratories, Burlingame, CA) and visualization with a peroxidase-based ABC detection system (Dako, Hamburg, Germany). To determine the degree to which the blood-brain barrier (BBB) was disrupted, the sections were incubated with a sheep anti-albumin antibody (1 : 300; Abcam, Cambridge, UK) that was detected by a biotinylated rabbit anti-sheep antibody (1 : 300; Southern Biotech, Birmingham, USA).

### 2.4. Flow Cytometry

All antibodies and reagents were obtained from BD Biosciences unless otherwise indicated: anti-CD3*ε* (145-2C11), anti-CD4 (RM4-5), anti-CD8*α* (53-6.7), anti-CD11a/LFA-1 (2D7), anti-CD25 (7D4), and anti-FoxP3 (FJK-16s, eBioscience). The antibodies were directly labeled with FITC, PE, PerCP, PE-Cy7, Cy5, APC, or APC-Cy7. Stainings were performed as previously described [10] and analyzed using a FACSCanto II or FACS Aria SORP device (BD Biosciences) in combination with FlowJo software.

### 2.5. CFSE Labeling and Transfer

T cells isolated from the spleens of C57Bl/6 mice constitutively expressing RFP were purified using a Pan-T-cell isolation kit (STEMCELL Technologies, Grenoble, France) and labeled with CFSE as previously described [15]. 1 × 10^7^ cells were adoptively transferred i.v. into C57Bl/6 mice that had been treated three times with 100 mg/kg MP, 100 mg/kg Dex, or PBS as a control and in which EAE had been induced on the second day of drug administration. Ten days later, spleen and lymph node cells from the recipient mice were analyzed by flow cytometry.

### 2.6. Statistical Analysis

Analysis was routinely performed by Mann-Whitney *U* and the unpaired *t*-test (Microsoft Excel and Graph Pad Prism Version 4). Data are depicted as the mean ± SEM; *P* > 0.05 was considered as nonsignificant (n.s.); *P < 0.05, **P < 0.01. To determine differences referring to the disease course, the whole curves rather than individual time points were compared between experimental groups. Strictly speaking, statistical analysis was performed from the day after the first treatment until the end of the observation period.

## 3. Results

**Figure 1:**
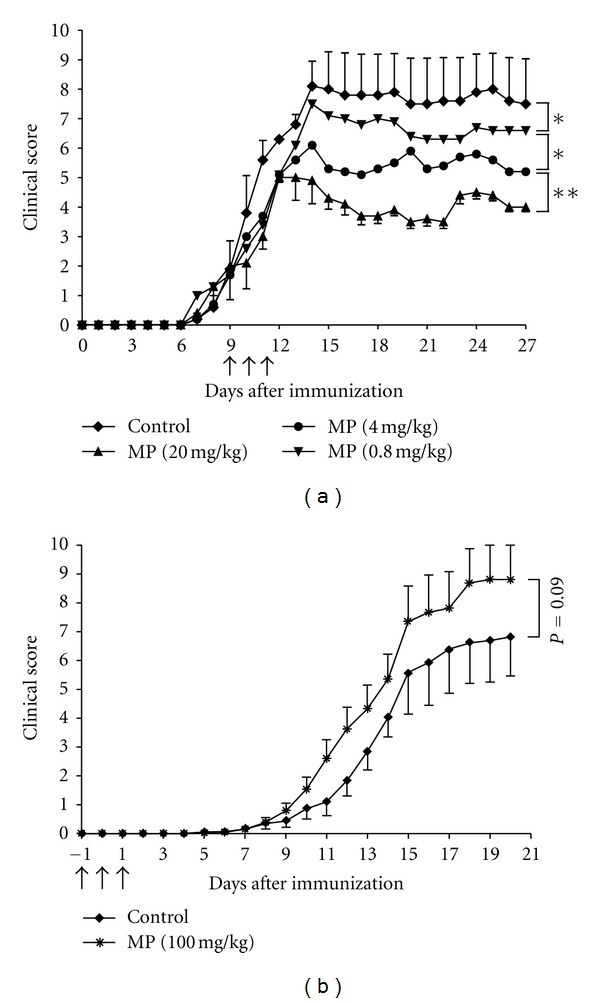
MP ameliorates EAE when applied in a therapeutic setting but aggravates it in a preventive setting. (a) EAE was induced in C57Bl/6 mice by immunization with MOG_35-55_ and treated with different doses of MP for three consecutive days starting at an average score of 2-3 (marked by arrows). *n* = 5 for each group. All values are depicted as mean ± SEM; error bars for the two intermediate dose groups are omitted for reasons of clarity. Statistical analysis: days 10–27 after immunization (*P < 0.05, **P < 0.01). (b) In a preventive setting, 100 mg/kg MP was administered to C57Bl/6 mice on days −1, 0, and +1 of immunization (marked by arrows). *n* = 8 for each group. All values are depicted as mean ± SEM. Statistical analysis: days 7–20.

We had previously reported that Dex ameliorates EAE in a dose-dependent manner when applied after the appearance of the first disease symptoms [10] and that it has a superior efficacy compared to an equimolar dose of MP [13]. Nevertheless, MP rather than Dex is the most widely used GC in the treatment of MS patients. Hence, we further investigated the effects of MP using the MOG_35-55_-induced EAE model in C57Bl/6 mice. When administered to mice with established EAE, the therapeutic efficiency of MP declined in a dose-dependent manner but still had a positive influence on the disease severity even at the lowest dose of 0.8 mg/kg (Figure 1(a)).

**Figure 2:**
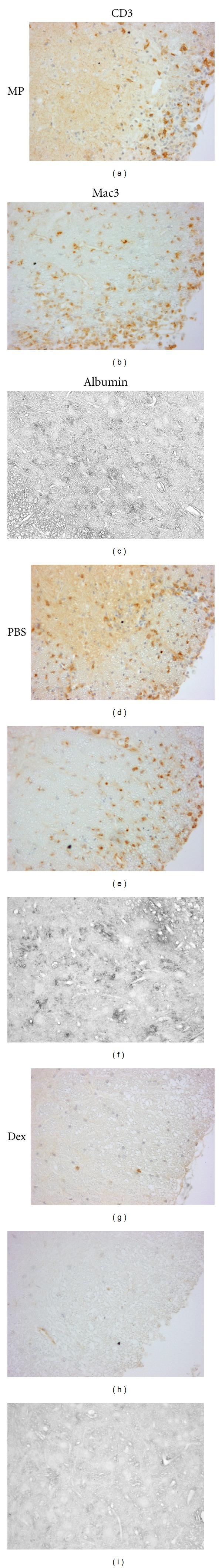
Immunohistochemical analysis of EAE following preventive GC administration. Immunohistochemical stainings are depicted for infiltrating T cells (left), macrophages/monocytes (middle), and albumin as marker of blood-brain-barrier integrity (right) in the lumbar spinal cord of one representative mouse of each treatment group (top: 100 mg/kg MP, middle: control, bottom: 100 mg/kg Dex) sacrificed on day 12. Magnification = 200x.

Dex administration around the time of immunization delays the onset of EAE and strongly diminishes the clinical symptoms [10]. Surprisingly, a similar treatment with 100 mg/kg MP starting on day −1 for three consecutive days had no beneficial effect on EAE and even aggravated the disease (Figure 1(b)). This unexpected finding was confirmed by histological analysis at day 12, revealing comparable numbers of infiltrating T cells and macrophages in the CNS of control mice as well as mice treated with 100 mg/kg MP (Figure 2). Moreover, the BBB was disrupted to a similar degree in both experimental groups (Figure 2). This is in strong contrast to the treatment of EAE with Dex, which almost completely prevents the manifestation of histopathological features of the disease (Figure 2).

**Figure 3:**
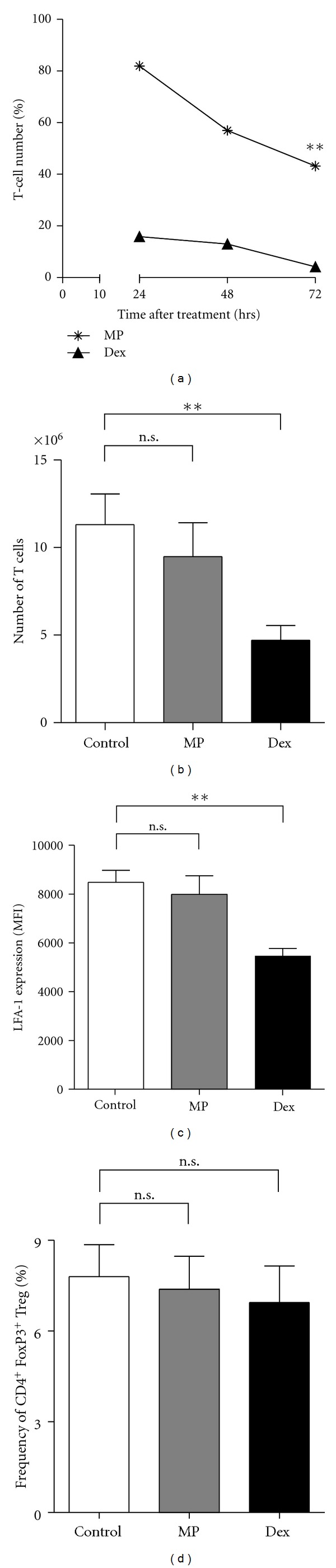
Flow cytometric analysis of peripheral T cells after preventive administration of GCs. (a) C57Bl/6 mice were treated three times with 100 mg/kg MP or Dex. Leukocytes were isolated from the spleens 24 hrs after the first, second, or third injection, stained for CD4 and CD3, and analyzed by flow cytometry. *n* = 2 (24 hrs/48 hrs), *n* = 5 (72 hrs). Statistical analysis for day 3 was performed using the unpaired *t*-test (**P < 0.01). (b) C57Bl/6 mice were treated three times at days −1, 0, and 1 with 100 mg/kg MP, 100 mg/kg Dex, or PBS as a control. At day 0, they were immunized with MOG_35-55_ according to the standard protocol. 12 days after immunization, leukocytes were isolated from the spleens, and absolute T-cell numbers were calculated based on flow cytometric analysis after staining for CD4 and CD8. *n* = 6. Statistical analysis was performed using the unpaired *t*-test (**P < 0.01). (c) The relative expression level of LFA-1 was determined after three injections of MP or Dex in splenic T cells using the same mice as in (A). The mean fluorescence intensity (MFI) 24 hrs after the last injection is depicted; *n* = 5 for each group. All values are depicted as mean ± SEM. Statistical analysis was performed using the unpaired *t*-test (**P < 0.01). (d) The percentage of FoxP3^+^ CD4^+^ CD25^+^ splenic T cells amongst all CD4^+^ T cells was determined 24 hrs after the last injection by flow cytometry; *n* = 5 for each group. All values are depicted as mean ± SEM. Statistical analysis was performed using the unpaired *t*-test.

In search of possible explanations for the observed discrepancy between the effects of preventive Dex and MP treatment, we injected mice on three consecutive days with 100 mg/kg Dex or MP followed by flow cytometric analysis of the peripheral T cells. While Dex induced a rapid and almost complete depletion of T cells in spleens and lymph nodes within three days, the effect of MP was much less pronounced (Figure 3(a)). The observed effect was long lasting, since absolute T-cell numbers in spleen (Figure 3(b)) and lymph nodes (data not shown) returned to normal levels on day 12 after preventive MP treatment, whereas they remained significantly diminished after Dex therapy. Dex also reduced the surface expression of the cell adhesion molecule LFA-1 by about half, whereas MP had no significant effect on LFA-1 expression (Figure 3(c)). Neither of the two drugs significantly affected the percentage of regulatory T cells (Figure 3(d)), ruling out the possibility that their preferential elimination by MP was responsible for the exaggerated disease course.

**Figure 4:**
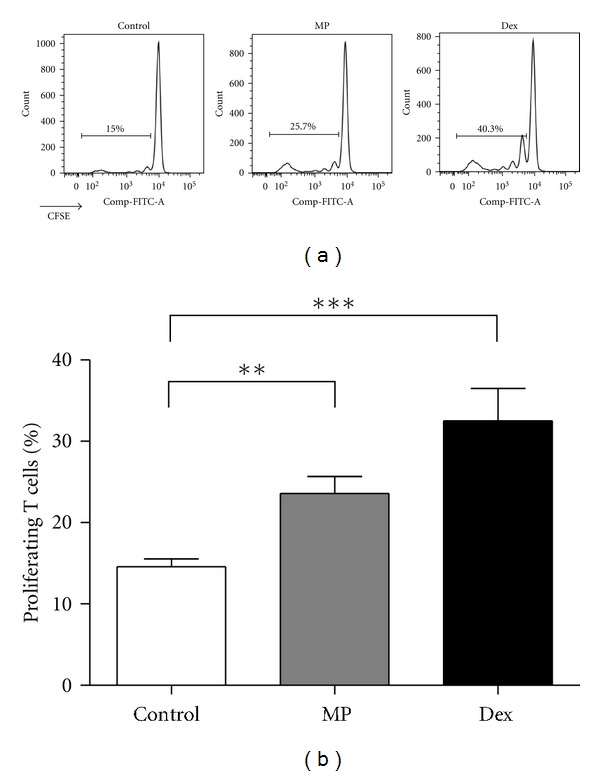
Homeostatic proliferation of transferred T cells after MP administration. C57Bl/6 mice were immunized with MOG_35-55_ peptide and treated with 100 mg/kg MP, 100 mg/kg Dex, or PBS as a control on days −1, 0, and 1. One day after the last treatment, 1 × 10^7^ purified CFSE-labeled T cells from RFP expressing mice were adoptively transferred, and ten days later, spleens and lymph nodes were analyzed by flow cytometry. (a) Representative histograms of lymph node total RFP^+^ T cells from PBS-(left), MP-(middle) or Dex-(right) treated mice are shown. (b) A quantification of the percentages of proliferating transferred T cells in the inguinal lymph nodes is depicted. *n* = 3-8 for each group. Statistical analysis was performed using the unpaired *t*-test (**P < 0.01, ***P < 0.001).

Preventive Dex administration impairs the priming of MOG_35-55_-specific T cells by efficiently deleting peripheral T cells via induction of apoptosis [10]. In contrast, MP treatment had a less potent proapoptotic activity than Dex (Figure 3(a)), which could be expected to foster homeostatic T-cell proliferation after cessation of the treatment. To test this hypothesis, mice were treated with GCs or PBS on three consecutive days starting one day before immunization. CFSE-labeled naïve T cells, which were purified from RFP-expressing mice to allow their tracking, were transferred on the day after the last injection of GCs. Flow cytometric analysis 10 days later revealed that the T cells had indeed started to proliferate in MP-treated mice, an effect which was significantly less pronounced in control mice (Figures 4(a) and 4(b)). This observation suggests that MP given in a preventive manner stimulates the immune response through augmenting homeostatic proliferation, thereby leading to the expansion of autoreactive T cells and thus eventually aggravating EAE. Preventive treatment of the mice with Dex resulted in an even more vigorous proliferation of the transferred T cells (Figures 4(a) and 4(b)).

## 4. Discussion

High-dose MP pulse therapy is widely used in the clinic to treat inflammatory conditions including autoimmune diseases. In MS, this regimen is still the measure of choice in the clinical management of acute relapses [1]. Furthermore, MP pulse therapy is given to prevent relapses in the postpartum period [16] and after cessation of natalizumab treatment [17]. In some clinical trials, MP therapy is also combined with classical immunomodulators [18, 19]. Given this widespread clinical importance of MP in MS therapy, we investigated its characteristics in more detail using the MOG-induced EAE model. Despite being less potent than Dex [13], we found that MP exerts a clear therapeutic effect in this model across a large range of dosages when applied after the onset of the disease. We and others have demonstrated that the potency of MP in the treatment of EAE can be further enhanced by encapsulating the drug into liposomes [13, 20, 21]. In a recent report, MP had been shown to reverse MOG-induced EAE in C57Bl/6 mice completely [22]. However, the dosage, route of delivery (orally versus i.p.), and time of application in the aforementioned work significantly differed from our protocol which might account for the observed discrepancy in the degree of disease suppression. In another study, MP had been therapeutically applied to Wistar rats in which EAE was induced by immunization with guinea pig spinal cord homogenate. In this case, MP also suppressed the disease which, however, could not be further potentiated by using dosages equivalent to the one we used here [23]. In contrast, if applied at lower doses, MP also exerted a dose-dependent effect in another rat EAE model [24].

Our previous studies had shown that Dex administered around the time of immunization significantly delays the onset of EAE and reduces its severity [10]. We now surprisingly found that the clinically more relevant MP, although also effective in the treatment of an established disease, worsened EAE when applied in a preventive setting. One explanation for this unexpected finding would be a different effect of Dex from MP on regulatory T cells, which are known to affect the strength of autoimmune diseases [25, 26]. Nonetheless, in our setting, we did not observe any effect of either of the two drugs on the percentage of these cells. We rather found that Dex is much more potent in reducing peripheral T-cell numbers in comparison to MP. While Dex almost completely deleted the T lymphocytes within three days, about half of them remained unaffected after MP administration. We had previously found that preventive Dex therapy resulted in delayed disease onset and reduced severity [10] and hypothesized that this beneficial effect of Dex in the preventive setting is a consequence of its potent proapoptotic activity ensuring a dramatic reduction in the precursor frequency of antigen-specific T cells reaching a level at which priming is severely impaired. Although both preventive Dex and MP therapy evoked homeostatic T-cell proliferation, the numbers of T cells after Dex application were decreased to such low numbers that priming of antigen-specific T cells could no longer occur efficiently. In contrast, preventive MP treatment only mildly reduced the number of peripheral T cells, thus leaving enough precursor cells in order for priming to take place. This notion is further underscored by our finding that 12 days after immunization absolute T-cell numbers were still significantly reduced in the spleen and lymph nodes in the case of preventive Dex treatment, whereas they had returned to normal levels after preventive MP treatment. Therefore, under conditions where T cells are mildly depleted for a limited period of time as is achieved by preventive MP therapy, T-cell priming is augmented rather than abolished since the homeostatic proliferation leads to the expansion of antigen-specific precursor cells. An alternative explanation for the aggravation of EAE after preventive MP treatment could potentially be unwanted side effects due to the ultrahigh MP dose used in these experiments. This, however, seems rather unlikely since the same ultrahigh dose resulted in amelioration of the disease when applied in a therapeutic setting [13], and also a lower dose of MP did not protect the mice from getting EAE in the preventive setting (data not shown). Even though our basic observation was already described for a rat model of EAE induced by immunization with spinal cord homogenate [27], there was no explanation for this finding, and our analyses now add new insight into the underlying mechanisms of this unexpected phenomenon.

It is reported that stress is a risk factor in multiple sclerosis and triggers acute relapses (for review, see [28]). Furthermore, it is known that stress enhances the release of hormones and neurotransmitters including corticosteroids [29]. Thus, it can be speculated that this higher level of endogenous corticosteroids in response to stress could also impact on the immune system by an enhanced apoptosis induction of peripheral lymphocytes, resulting in homeostatic proliferation and thereby activation of self-reactive T cells.

In summary, we believe that the feature of MP described in this paper might have implications for its therapeutic success in the clinic and should be followed up in translational settings and in the current clinical practice.
